# Leveraging social media to increase lung cancer screening awareness, knowledge and uptake among high-risk populations (The INSPIRE-Lung Study): study protocol of design and methods of a community-based randomized controlled trial

**DOI:** 10.1186/s12889-023-15857-8

**Published:** 2023-05-26

**Authors:** Lisa Carter-Bawa, Smita C. Banerjee, Robert S. Comer, Minal S. Kale, Jennifer C. King, Katherine T. Leopold, Patrick O. Monahan, Jamie S. Ostroff, James E. Slaven, Francis Valenzona, Renda Soylemez Wiener, Susan M. Rawl

**Affiliations:** 1 Center for Discovery & Innovation, Hackensack Meridian Health, Cancer Prevention Precision Control Institute, 111 Ideation Way, Nutley, NJ 07110 USA; 2 Memorial Sloan Kettering Cancer Center, New York, NY USA; 3 Indiana University Purdue University, Indianapolis, IN USA; 4 Icahn School of Medicine at Mount Sinai, New York, NY USA; 5 GO2 for Lung Cancer, Washington, DC USA; 6 Hackensack Meridian School of Medicine, Nutley, NJ USA; 7 Indiana University School of Medicine, Indianapolis, IN USA; 8 Center for Healthcare Organization & Implementation Research, VA Boston Healthcare System, Boston, MA USA; 9 The Pulmonary Center, Boston University, Boston, MA USA; 10 Indiana University School of Nursing, Indianapolis, IN USA

**Keywords:** Screening, Lung cancer, Social media, Decision support, Tailored communication, Facebook targeted advertisement

## Abstract

**Background:**

Lung cancer is the leading cause of cancer death for both men and women in the United States. The National Lung Screening Trial (NLST) demonstrated that low-dose computed tomography (LDCT) screening can reduce lung cancer mortality among high-risk individuals, but uptake of lung screening remains low. Social media platforms have the potential to reach a large number of people, including those who are at high risk for lung cancer but who may not be aware of or have access to lung screening.

**Methods:**

This paper discusses the protocol for a randomized controlled trial (RCT) that leverages FBTA to reach screening-eligible individuals in the community at large and intervene with a public-facing, tailored health communication intervention (*LungTalk*) to increase awareness of, and knowledge about, lung screening.

**Discussion:**

This study will provide important information to inform the ability to refine implementation processes for national population efforts to scale a public-facing health communication focused intervention using social media to increase screening uptake of appropriate, high-risk individuals.

**Trial registration:**

The trial is registered at clinicaltrials.gov (#NCT05824273).

## Background

Lung cancer kills more people annually than breast, cervical, colorectal, and prostate cancers combined [1]. Lung screening with annual low-dose computed tomography (LDCT) reduces lung cancer-related mortality by identifying lung cancer at earlier, more treatable stages [2–4]. *However, population-level screening efforts are only effective when eligible, high-risk individuals are aware and engaged.* In 2015, the Centers for Medicare and Medicaid agreed to cover lung screening in response to the 2013 U.S. Preventive Services Task Force (USPSTF) Grade B recommendation for individuals aged 55 to 80 years with a 30-pack-year smoking history who currently smoke or have quit within the past 15 years [2, 3, 5]. In response to new scientific evidence, updated recommendations have decreased the minimum screening eligible age to 50 years and pack-year history to 20 [6]. As part of Medicare’s coverage, in order for lung screening to be reimbursed, a shared decision-making and counseling visit must be conducted with one or more patient decision aids [5]. Medicare’s unprecedented policy and coverage mandate fostered a unique opportunity for advancing understanding of the shared decision-making process for lung screening.

Although lung screening is recommended by the USPSTF [2], has the potential to detect lung cancer at earlier, more treatable stages, has a 20% lung cancer-related mortality reduction in individuals who smoke long-term [3, 4] and is covered by Medicare and other health insurers [5] population uptake has been abysmal. Nearly a decade after the USPSTF recommended lung cancer screening, less than 5% of screening-eligible Americans have been screened [7]. Screening-eligible individuals are generally unaware lung screening exists [8–11], and screening-eligible individuals in the U.S. do not screen – *when they are aware* – because of barriers to screening [8, 10]. Given that high-risk individuals are generally not aware that lung screening exists, it is essential to employ new and novel community-focused communication strategies to increase awareness about lung screening so as to reach high-risk, screening-eligible individuals. Social media offers untapped opportunities to address the lack of awareness and knowledge about lung screening and thereby reach high-risk, screening-eligible individuals and increase screening adoption. As of 2023, individuals aged 65 and older are the fastest growing demographic group on Facebook and use of this social media platform among individuals born in or before 1945 has nearly doubled in the past three years. Further, among the 2.7 billion Facebook users, over 32 million are age 50 years and older – the age range for lung screening eligibility [11]. Because Facebook has the ability to target advertisements to individual users by key demographic and interest areas within their profile, Facebook-targeted advertisement (FBTA) offers an ideal social media platform to reach and deliver a public-facing, tailored health communication and decision support intervention to increase awareness of, and knowledge about, lung screening among those most at risk.

To facilitate awareness of the option to screen for lung cancer and support meaningful patient-clinician discussions about screening, effective communication strategies are needed to prepare patients to initiate (“Ask your doctor”) and to *have* these important discussions with their clinician. To foster both, our team developed *LungTalk *[8], a novel computer-tailored health communication and decision support tool to (1) increase awareness and knowledge about lung screening; (2) decrease perceived barriers to screening by addressing misinformation; (3) increase occurrence of a patient-clinician discussion about lung screening; and (4) increase screening rates among individuals whose decision after a shared decision-making discussion with their clinician is to screen. Given that new and novel ways to increase awareness and knowledge about lung screening and adoption in high-risk populations are essential to support effective population-based lung screening implementation, we seek to better understand how to raise awareness about lung screening.

Historically low levels of public trust in expert entities such as government, news media, and the healthcare system as well as growing awareness of the new cancer information ecosystem led us to consider social media as a novel platform for cancer communication. FBTA has been successfully used by our team and others to recruit individuals into research studies [10, 12–15], and provides “precision marketing” – sending the right message content to the right person at the right time via the right channel.

Early health communication tools and decision aids for lung screening have primarily focused on calculating personal risk for the development of lung cancer and subsequent recommendations to screen based upon that risk [16–18]. These tools range in level of complexity and delivery including pamphlets and brochures in print, web-based information, videos, educational scripts, and computer programs [16–21]. These tools can also be deployed in multiple formats such as by mail, telephone, in person and via the internet and have been found to be effective [16–21]. *LungTalk* tailors messages based on smoking status, perceived barriers, and priorities for discussion in the patient-clinician encounter, allowing *LungTalk* to address individualized issues that are personally relevant to the lung screening decision. Ultimately, to support shared decision-making in lung screening, it is critical that health communication tools and decision aids about lung screening go beyond assessing risk and tailor messages based on multiple salient variables that may be personally relevant to the patient. *LungTalk* can be used as a public-facing health communication tool at multiple time points: prior to entrance into the healthcare system, during a clinic visit, and post-visit to support the decision to screen, or not, for lung cancer. In addition, *LungTalk* goes beyond risk assessment screening education to increase perceived benefits and self-efficacy and reduce perceived barriers in order to move a screening-eligible individual forward in stage of adoption for lung screening [8]. Finally, *LungTalk* is theoretically grounded, which increases our ability to determine what components of the intervention are driving behavior change [8, 22].

Our overall objective in this study is to test the effectiveness of: 1) leveraging a well-established, social media-based platform (FBTA) to target screening-eligible individuals in the community and 2) a novel, tailored health communication and decision support intervention related to lung screening (*LungTalk*). Our central hypothesis is two-fold: 1) FBTA will be a successful platform to reach high-risk individuals who have not previously undergone or sought lung screening; and 2) tailored lung screening information compared to non-tailored information will increase knowledge and improve health beliefs about screening and subsequent screening uptake. This paper discusses the protocol for a randomized controlled trial (RCT) that leverages FBTA to reach screening-eligible individuals in the community at large and intervene with a public-facing, tailored health communication intervention (*LungTalk*) to increase awareness of, and knowledge about, lung screening.

## Methods

### Overview

The INSPIRE-Lung Study is designed as a randomized, controlled, community-based trial with two parallel groups and a primary endpoint of lung screening uptake by 6 months post intervention. Randomization will be performed as block randomization with a 1:1 allocation. See Table 1 for Trial Registration Data and Table 2 for SPIRIT Flow Diagram of Participant Timeline. This study has two components: (1) to assess the ability of FBTA to *reach* high-risk individuals eligible for lung screening; and (2) to examine the comparative-effectiveness of *LungTalk* and a non-tailored lung screening information video in a national sample of screening-eligible, community-based individuals using an RCT design. *Reach* is defined as the absolute number, proportion, and representativeness of participating individuals assessed for lung screening knowledge, awareness and uptake, and reasons why or why not. *Effectiveness* is defined as increased knowledge, decreased perceived barriers to lung screening, occurrence of a patient-clinician discussion about the option to screen, and screening uptake, if the decision is to screen. Potential moderators of effectiveness (i.e., smoking status, gender, age, family history of lung cancer, provider recommendation, stigma, mistrust, fatalism, fear, worry, lung screening health beliefs) will also be assessed. This study was approved by the Institutional Review Board of Hackensack Meridian Health (IRB Protocol #: Pro-2022–0860). In addition, all methods will be performed in accordance with the guidelines and ethical principles that are fundamental to human subject protection and electronic written informed consent will be obtained online from all study participants.

**Table 1:** Trial registration data

Data Category	Information
Primary registry and trial identifying number	ClinicalTrials.gov NCT05824273
Date of registration in primary registry	10 April, 2023
Source of monetary or material support	National Cancer Institute R01CA263662
Primary sponsor	Hackensack Meridian Health
Collaborator	National Cancer Institute (NCI)
Contact for public queries	Lisa Carter-Bawa, PhD, MPH, APRN, ANP-C, FAAN [lisa.carterbawa@hmh-cdi.org]
Contact for scientific queries	Lisa Carter-Bawa, PhD, MPH, APRN, ANP-C, FAAN, Center for Discovery & Innovation, Hackensack Meridian Health, Nutley, NJ, USA
Public title	The INSPIRE-Lung Study
Scientific title	Leveraging Social Media to Increase Lung Cancer Screening Awareness, Knowledge and Uptake in High-Risk Populations
Countries of recruitment	USA
Health problem studied	Lung cancer screening
Intervention(s)	Intervention: computer-tailored health communication and decision support tool (*LungTalk*) Comparator/attention control: non-tailored lung cancer screening video
Key inclusion and exclusion criteria	Ages eligible for study: 50–80 years; Sexes eligible for study: both; Accepts Healthy Volunteers: Yes
	Inclusion criteria: ≥ 20 pack-year smoking history, individuals who currently smoke or quit smoking within the past 15 years
	Exclusion criteria: have previously undergone LDCT for early detection of lung cancer, have a lung nodule or nodules that are currently being followed, have been diagnosed with lung cancer
Study type	Interventional
	Allocation: randomized; Intervention model: parallel assignment; Masking: none
Date of first enrollment	June 1, 2023 (anticipated)
Target sample size	500
Recruitment status	Not yet recruiting
Primary outcome(s)	Reach (reaching screening-eligible individuals via social media) Effectiveness of *LungTalk* Knowledge of Lung Cancer & Screening Screening Uptake (time frame: 6 months)
Key secondary outcomes	Occurrence of a Patient-Clinician Discussion Lung Cancer Screening Health Beliefs (perceived risk, perceived benefits, perceived barriers, self-efficacy)

**Table 2:** SPIRIT Flow Diagram

	**STUDY PERIOD**				
	**Enrollment**	**Allocation**	**Post-allocation**		**Close-out**
**TIMEPOINT**	***Baseline***	**0**	***1 week***	***6 months***	***7 months***
**ENROLLMENT:**					
**Eligibility screen**	X				
**Informed consent**	X				
**Allocation**		X			
**INTERVENTIONS:**					
***LungTalk***		X			
***Non-tailored LCS Video***		X			
**ASSESSMENTS:**					
*Knowledge: LCS; LCS Health Beliefs; Patient-Clinician Discussion*	X	X			
*Screening Uptake; Patient-Clinician Discussion about LCS*				X	X
*Stage of Adoption for LCS*			X	X	X

*LCS* lung cancer screening

### Study setting

We will leverage FBTA to recruit community-based lung screening-eligible individuals. Using the Centers for Disease Control and Prevention Smoking and Tobacco Use statistics [23], we chose states with a relatively high (e.g., 15.9% or greater) adult smoking rate representing all U.S. census regions. We will employ FBTA in five states including Indiana, Kentucky, Pennsylvania, Oklahoma, and Oregon. We chose these five states because they are geographically diverse across the U.S., their populations are racially and ethnically diverse, and they have moderate-to-high adult smoking rates increasing the likelihood of reaching screening-eligible individuals. We are partnering with the GO2 for Lung Cancer (GO2) to identify Centers of Excellence in Lung Cancer Screening (as designated by GO2) [24] to connect individuals who seek a screening referral request but do not have a primary care clinician. There are more than 800 Centers of Excellence in Lung Cancer Screening nationwide, and there are currently 126 in the five states in which we will conduct the study. These centers are well-established, well-connected, dedicated to high quality screening and care, and have strong collaborators with their local primary care networks, making them the ideal national partner to connect participants without a primary care clinician to one for facilitating the patient-clinician discussion about screening. They can also link individuals to primary care clinicians regardless of insurance status through federally qualified health centers and community health centers fostering access to high quality screening for low income and other vulnerable subpopulations.

### Sample eligibility criteria

Eligibility criteria mirror the current USPSTF lung screening guidelines: 1) aged 50 to 80 years; 2) ≥ 20-pack-year smoking history; 3) individuals who currently smoke or quit smoking within the past 15 years [6]. Participants will be excluded if they are non-English speaking, have previously undergone LDCT for early detection of lung cancer, have a lung nodule or nodules that are currently being followed, or if they have been diagnosed with lung cancer.

### Recruitment rationale and procedures

**Fig. 1:**
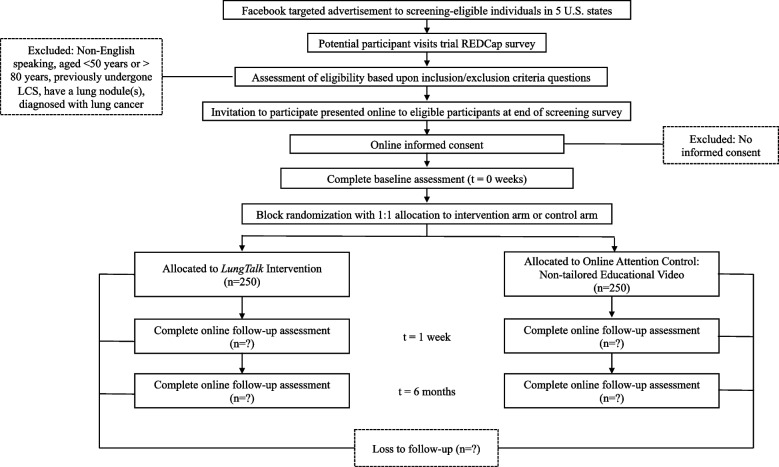
CONSORT flow diagram for INSPIRE-Lung Trial

We will use a highly successful recruitment strategy via FBTA [25] to recruit 500 screening-eligible individuals from Indiana, Kentucky, Pennsylvania, Oklahoma, and Oregon. See Fig. 1 for CONSORT diagram. The Facebook user’s interest list includes a wide range of details a user can select when setting up and/or maintaining their profile that they have an interest in such as groups, hobbies, lifestyle choices, behaviors, points of view, specific organizations and more. This allows us to purposively sample people who are age 50 years and older, indicate smoking or smoking cessation as an interest and reside in a particular state, city, or zip code. Using this approach, as we have in prior studies [8, 10, 26], we will target our advertisement on Facebook using the following keywords: *cigarette, tobacco, nicotine replacement therapy, nicotine gum, electronic cigarette, smoking, vaping*. Guided by the safety and monitoring guidelines for researchers using social media [27, 28], our approach includes design and close monitoring of the FBTA to ensure all methodologic and ethical standards are upheld. Currently, we have a potential reach of 550,000 potentially screening-eligible individuals in the 5 states above. As an example, with a $5,000 recruitment budget and an ad campaign that runs for 14 days, Facebook analytics estimate that 28,000 to 82,000 people per day will see the ad in their daily news feed in the five states and 262 to 758 unique Facebook users will click on the embedded eligibility survey link within the advertisement on a daily basis.

### Description of the intervention

**Fig. 2:**
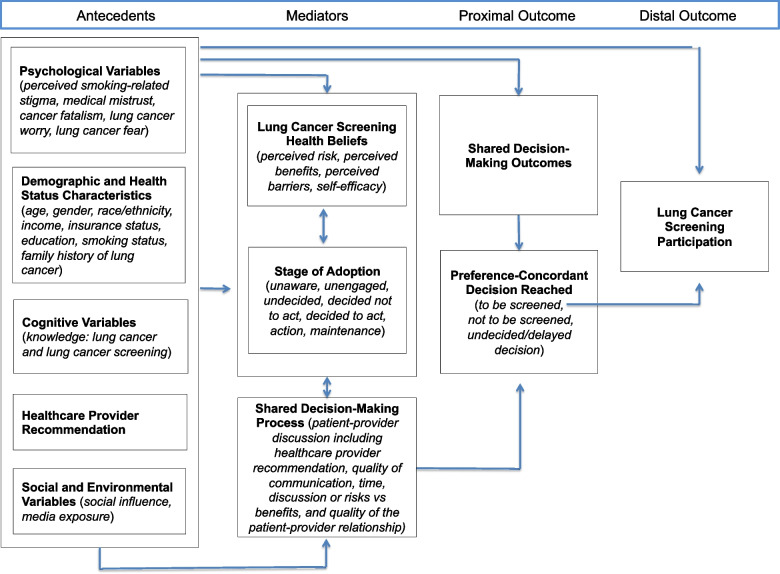
Conceptual model for lung cancer screening participation

Individuals who enroll into the study will be randomized to one of two arms: *LungTalk* or the non-tailored lung screening educational video. *LungTalk* is a computer-tailored health communication and decision-making tool that is theoretically grounded in the Conceptual Model on Lung Cancer Screening Participation [22]. See Fig. 2. This model links the Health Belief Model to the Precaution Adoption Process Model and includes key psychological variables (e.g., stigma, mistrust, fatalism, fear and worry) as factors that may influence an individuals’ decision to screen, or not, for lung cancer [22]. The tool as a whole serves as a cue to action for a screening-eligible individual to engage in a discussion with their clinician about the option to screen, or not, for lung cancer.

*LungTalk* is an interactive computer program that takes approximately 8 to 12 min to complete depending upon specific tailoring variables selected by the user and includes embedded audio, video and animation segments with tailoring algorithms for scripts presented from a master content library [8]. *LungTalk* tailors initially on smoking status. Early in the program, the user is asked to indicate if they currently smoke cigarettes or if they quit smoking and subsequent content is aligned with an individual’s current smoking status. In addition, *LungTalk* tailors on the top three barriers to lung screening identified in our prior research testing the Conceptual Model for Lung Cancer Screening Participation (R15 CA208543) [22]. Those barriers are: (1) cost/insurance issues; (2) worry; and (3) not receiving a recommendation from a healthcare clinician. After viewing an embedded video of how a lung scan is performed, the user will be asked if they perceive cost/insurance, worry, or lack of receiving a clinician recommendation as a barrier to getting screened. At this point, the user is able to choose none, one, two, or all three barriers. Based upon the user’s responses, *LungTalk* will play a brief video that includes messaging to address the perceived barrier. *LungTalk* concludes by offering the option of saving or printing a tailored summary at the end for individuals to use as a discussion prompt with their clinician [8]. This print-out highlights key points related to lung health and screening, tailored by smoking status, offers question prompts to initiate a discussion with their clinician, and tailors the question prompts based upon questions that remain important to the user that they wish to discuss further with their clinician [8]. Messages in *LungTalk* are presented at an 8th grade reading level, and in consideration of different ways people like to learn, the content is narrated as well as presented as key text on the screen.

The *comparator/attention control condition* is a non-tailored 5-min video from the GO2 for Lung Cancer about lung screening designed for the lay individual [29]. This video was chosen as the comparator to serve as an attention control condition for the nonspecific effects of the intervention by balancing attention, treatment contact, and delivery channel so that a precise test of the hypothesized active component (the tailoring in *LungTalk*) of the intervention comparator can be made. Attention control conditions require two components – clinical attention and a therapeutic benefit; choosing the GO2 for Lung Cancer’s lung screening video meets both requirements.

### Intervention delivery

Intervention delivery will occur online. Eligible participants will be randomly assigned to either the intervention (*LungTalk*) or attention control (non-tailored educational video) after completion of the baseline survey with a 1:1 allocation as per a computer-generated randomization schedule stratified by smoking status (current or former). After recruitment, participants will be redirected to the REDCap platform to complete the informed consent and baseline survey. Participants then will be randomized and directed to their assigned intervention condition (i.e., *LungTalk* or attention control).

### Data collection

This study is focused on both the implementation of a social media-based communication platform to increase awareness about lung screening as well as the effectiveness of a tailored health communication and decision support tool (*LungTalk)*. For the implementation of a social media-based communication platform, the primary outcome is reach. For the effectiveness of *LungTalk*, the primary outcome is screening uptake. All investigators (principal and co-investigators) will be given access to the cleaned de-identified data sets. Project data sets will be housed on the Hackensack Meridian Health password-protected server and a file transfer protocol site created for the study. All data sets will be password protected. To ensure confidentiality, data dispersed to study team members will be blinded of any identifying participant information.

#### Assessment of reach

Inherent in FBTA are a number of standard analytics [30] that will facilitate our ability to assess the number, proportion, and representativeness of individuals who are exposed to both health communications about lung screening. Using the FB analytics component [30] of our FBTA, our assessment plan will measure the total reach of the FBTA to increase awareness of the option to screen for lung cancer among screening-eligible individuals. Quantitative data collected from FB analytics during the recruitment campaign will assess reach by detailing: 1) reach; 2) link clicks; and 3) impressions. Reach in FB analytics is defined as the number of people who saw the FBTA at least once [30]. This can be further analyzed by hour, day, specific number of days, week, and campaign length. In addition, reach can be further stratified by specific location (i.e., state, city, town, county, zip code) [30].

#### Assessment of effectiveness

Our assessment plan will compare the effectiveness of a tailored (*LungTalk*) health communication and decision support tool versus non-tailored health communication tool delivered online to improve: 1) total knowledge about lung screening; 2) lung cancer screening health beliefs; 3) occurrence of a patient-clinician discussion about lung screening; and 4) screening uptake. After enrollment, we will conduct a baseline survey using REDCap Survey with validated measures [31] used in our prior work assessing knowledge, lung cancer screening health beliefs, occurrence of a patient-clinician discussion about lung screening and stage of adoption for lung screening among 500 screening-eligible individuals in 5 states representing socioeconomically, ethnically, and geographically diverse locations. Grounded in the Precaution Adoption Process Model, stage of adoption is defined as seven stages an individual may be classified when presented with a health decision: (1) unaware, (2) aware but unengaged, (3) undecided, (4) decided not to act, (5) decided to act, (6) action, and (7) maintenance. Stratified by smoking status, participants will then be randomized to *LungTalk* or attention control. One week after delivery of the intervention, participants will complete an online follow-up survey to assess changes in knowledge, lung cancer screening health beliefs, occurrence of a patient-clinician discussion about lung screening, and screening uptake. At six months, participants will complete another online survey to assess occurrence of a patient-clinician discussion about lung screening and screening uptake. See Table 3 for Measures of Assessment.

**Table 3:** Measures of assessment

To assess the ability of FBTA to reach high-risk individuals eligible for lung screening					
**Constructs**	**Assessment or Measure**				
Reach	Total # of people who saw the FBTA at least once				
Link Clicks	Total # of clicks on the link within the FBTA that led to the REDCap survey platform of the study				
Impressions	Total # of times the FBTA was on screen (may include multiple views of the ad by the same person/people)				
To examine the comparative-effectiveness of *LungTalk* and a non-tailored lung screening information video in a national sample of screening-eligible, community-based individuals using an RCT design					
			**Timeline of Assessment**		
**Constructs**	**Assessment or Measure**	**# of Items**	**Baseline**	**1 wk**	**6 mo**
Knowledge	Knowledge: Lung Cancer Screening	9	X	X	
Perceived Risk	Perceived Risk of Lung Cancer Scale [32]	3	X	X	
Perceived Benefits	Perceived Benefits of Lung Cancer Screening Scale [32]	6	X	X	
Perceived Barriers	Perceived Barriers to Lung Cancer Screening Scale [32]	17	X	X	
Self-Efficacy	Self-Efficacy for Lung Cancer Screening Scale [32]	9	X	X	
Occurrence of Patient-Clinician Discussion	Self-report of Occurrence of a Patient-Clinician Discussion about Lung Cancer Screening	1	X	X	X
Screening Uptake	Self-report via the stages of adoption algorithm for screening with verification process	1	X	X	X

In order to mimic real-world implementation of a social media campaign to increase screening uptake, participant incentives will not be offered for the baseline survey. However, after enrollment in the study and viewing the intervention to which the participant has been randomized, participants will learn that they will receive a monetary gift card upon completion of the follow-up surveys at two time points following intervention ($50 after 1-week survey post-intervention; $25 after 6-month survey post-intervention).

#### Retention

Several techniques to increase engagement in the study over the 6-month follow-up period will be employed such as: 1) communicating clearly the requirements of the study during the recruitment phase; 2) obtaining alternative contact information such as phone numbers (i.e., home, work, cell) and participant email address for follow-up; and 3) sending out an electronic newsletter to report the progress of the study.

### Data analysis and interpretation

#### Analysis of reach

Using data from the Facebook analytics collected during the targeted advertisement period, we will analyze reach in the following ways: (1) number and percentage of individuals age 50 and older who currently or formerly smoke in the population in which the advertisement is marketed; (2) percentage of eligible participants who agree to participate in the study; (3) compare differences between those participating and those not participating on smoking status (i.e., current vs. former), age, gender, geography and other key variables collected on the screening survey; (4) record reasons that participants refuse to participate in the study; (5) estimate attrition at 1 week and 6-month follow-up time periods; and (6) compare differences between those completing and those not completing the study on sociodemographic and health status variables, geography, baseline scores on knowledge, lung cancer screening health beliefs, and stage of adoption for lung cancer screening. Facebook generates analytics related to the advertisement and includes descriptive statistics such as proportions and means to assess reach, link clicks, and impressions, as described above, for the FBTA [30].

#### Analysis of effectiveness

Our analyses were defined a priori to address the study aims. We will use descriptive statistics such as means, standard deviations, and frequency distributions/distributional assumptions to examine data quality, identify patterns of missing and out-of-range values, and evaluate the assumptions of statistical tests. Specifically, we will examine all aspects of data quality to ensure statistical integrity and accuracy including: 1) data skewness, kurtosis, and parametric assumptions; 2) intention-to-treat (ITT) principles; 3) missing data considerations; and 4) control of overall alpha to avoid inflated experiment-wise Type-I error due to multiple statistical tests. Remediation of normal distribution assumption violations will be accomplished using methods such as data transformations (e.g., log or square root for positively skewed variables), Box-Cox family transformations, or kernel estimation techniques to determine the best-fitting parametric density [33], or other methods as appropriate. Assessment of internal consistency reliability of all scales will be carried out using the Cronbach’s alpha coefficient. We will apply the intention-to-treat (ITT) principle in handling missing data on screening uptake. A study participant will be coded as ‘no screening uptake’ unless otherwise verified by our participating sites. We will examine violations of the missing-at-random assumption. If missing is not completely at random, then covariates associated with missingness will be incorporated into data analysis to minimized. A related approach is the use of mixed-effects models, which is capable of handling binary as well as continuous outcomes [34], to use all available behavioral outcomes data since HLM does not carry out list-wise deletion by default, thus the statistical power loss due to missing data may be minimal. Additionally, missing outcome data (assuming up to 20% of the respondents will be unreachable at our three-month follow-up) as well as missing assessments may be amenable to imputation by several techniques that can handle both continuous and categorical missing data [35–37]. We may also use the Pattern-Mixture Model to examine whether or not missed follow-up assessments are associated with baseline characteristics with safeguards to minimize model overfit. Finally, to control for potentially inflated Type-I error rate due to multiple comparisons, analyses will incorporate a multiple comparisons method, such as a False Discovery Rate-controlling procedure, which is more powerful than simple Bonferroni corrections [37].

Total knowledge scale scores, Lung Cancer Screening Health Beliefs (total scale scores for perceived risk, perceived benefits, perceived barriers, self-efficacy), and stage of adoption are continuous variables. Screening referral request and screening uptake are dichotomous variables. Prior to group comparisons, measures will be described by timepoint, both overall and by group. Within-group changes will be assessed using the standardized response mean (SRM) effect sizes (mean change divided by SD of change). The primary outcome is lung screening uptake but forward movement in stage of adoption (i.e., the change score) will also be evaluated as a secondary analysis. For between-group comparisons, continuous outcome variables will be compared using two-sided independent-sample *t*-tests (accompanied by the standardized mean difference effect size, i.e., difference between group means divided by baseline SD) and dichotomous outcome variables will be compared using the Chi-square test, or two-sided Fisher’s exact test, if 20% or more cells have expected counts less than 5 (accompanied by the odds ratio effect size). In the case of differential attrition, as noted above, regression models will also be used to assess outcomes by adjusting for baseline covariates that differ significantly between participants who do and do not complete follow-up assessments. Moderators of intervention effectiveness will be assessed by regressing outcome variables (e.g., knowledge score) on randomization arm, the potential moderator, and an interaction term, where a significant interaction effect is indicative of moderation. Significant moderator effects will further be explored and described with stratified analyses.

#### Sample size justification and power analysis

With a sample size of *n* = 250 per intervention group, and assuming an ITT analysis (with a default of no screening uptake unless otherwise verified), we will be able to detect a difference between the *LungTalk* and the attention control intervention condition groups with an 81% statistical power if the difference in lung screening uptake is 31% in the *LungTalk* group compared to 10% in the attention control intervention condition group, in a test of independent proportions and a two-sided type-I error rate of 5%. This 31% versus 10% difference is based on preliminary data testing *LungTalk* in a sample of community-based screening-eligible individuals in Indiana in 2018 using the same ITT procedure. With a sample size of *n* = 250 per group and up to 20% missing assessment data (*n* = 200 available for analysis) on total knowledge scale and total perceived risk scale scores, we will have an 80% statistical power if the difference is d = 0.28 (in standardized effect size units, or Cohen d), in an independent-sample t-test with a two-sided type-I error rate of 5%. A 0.25 effect is considered a ‘small’ effect size in psychology-based research, thus a conservative estimate of the statistical evidence that can be supported in our study design.

We expect to start enrollment in June 2023 and conclude the study in the Summer of 2027. Data analysis will be completed by December 2027.

## Discussion

To date, researchers have focused on the implementation of shared decision-making in lung screening using various decision aids at the point of healthcare delivery. We are challenging the current status quo by shifting the focus of outreach and engagement back before the screening-eligible individual enters the healthcare system to identify effective communication platforms and interventions to increase lung screening awareness and knowledge. Upon study completion, we will have identified the reach of FBTA, and determined the effectiveness of a computer-tailored health communication and decision support tool intervention (*LungTalk*) using a social media campaign to increase screening awareness and uptake. Educating screening-eligible individuals about key factors related to lung screening at a population-based level by leveraging social media as a platform to reach the right people in order to implement this effort has the potential to enhance patient outcomes by: 1) increasing baseline knowledge; 2) decreasing misinformation; and 3) decreasing perceived barriers to screening. We expect that leveraging social media to increase awareness and knowledge will be an effective public-facing communication strategy for complex health topics such as lung screening. Equally important will be our ability to identify and engage vulnerable patient populations based upon precise targeting criteria. Ultimately, using a novel health communication strategy to tailor health messages based upon characteristics that are unique to the individual as well as leveraging social media to reach the target population and deliver this type of intervention are both innovative methods to engage high risk individuals [38–41]. These findings will be used to inform how public health campaigns in lung screening can be scaled to support increasing awareness and knowledge. This contribution is significant because the gap in the current state of the science in lung screening is population awareness and knowledge which has led to the abysmal rates of lung screening discussions with clinicians (< 10%) [11] and screening uptake (< 5%) [7].

### Protocol modifications

Modifications to the protocol which may have an impact on the conduct of the study, potential benefit of the patient or may risk to the participant, including changes of study objectives, study design, target population, sample sizes, study procedures, or significant administrative aspects will require a formal amendment to the protocol. Such amendment will be submitted for review by the Hackensack Meridian Health Institutional Review Board for approval.

## Data Availability

Not applicable.
